# Molecular Cloning of Alternative Splicing Variants of the Porcine *PML* Gene and Its Expression Patterns During Japanese Encephalitis Virus Infection

**DOI:** 10.3389/fvets.2021.757978

**Published:** 2021-11-23

**Authors:** Jingjing Zhu, Zhenyu Chen, Zhenglie Dai, Xiaolong Zhou, Han Wang, Xiangchen Li, Ayong Zhao, Songbai Yang

**Affiliations:** Key Laboratory of Applied Technology on Green-Eco-Healthy Animal Husbandry of Zhejiang Province, College of Animal Science and Technology, College of Veterinary Medicine, Zhejiang A&F University, Hangzhou, China

**Keywords:** porcine, *PML* gene, alternative splicing, clone, JEV

## Abstract

Promyelocytic leukemia (PML) protein is a crucial component of PML-nuclear bodies (PML-NBs). PML and PML-NBs are involved in the regulation of various cellular functions, including the antiviral immune response. The human *PML* gene can generate several different isoforms through alternative splicing. However, little is known about the porcine PML alternative splicing isoforms and their expression profiles during Japanese encephalitis virus (JEV) infection. In the present study, we cloned seven mature transcripts of porcine PML, all of which contained the same N-terminal sequence but differed in the C-terminal sequences due to alternative splicing. These seven transcripts encoded five proteins all of which had the RBCC motif and sumoylation sites. Amino acid sequence homology analysis showed that porcine PML-1 had relatively high levels of identity with human, cattle, and goat homologs (76.21, 77.17, and 77.05%, respectively), and low identity with the mouse homolog (61.78%). Immunofluorescence analysis showed that the typical PML-NBs could be observed after overexpression of the five PML isoforms in PK15 cells. Quantitative reverse transcription PCR (RT-qPCR) analysis showed significant upregulation of PML isoforms and PML-NB-associated genes (*Daxx* and *SP100*) at 36 and 48 h post-infection (hpi). Western blotting analysis indicated that the PML isoforms were upregulated during the late stage of infection. Moreover, the number of PML-NBs was increased after JEV infection. These results suggest that porcine PML isoforms may play essential roles in JEV infection.

## Introduction

Promyelocytic leukemia (PML) protein, also known as TRIM19, is a member of the tripartite motif (TRIM) protein family and was originally identified at the *t*(15;17) chromosomal translocation in acute promyelocytic leukemia (APL) (1). PML is critical for the formation of PML-nuclear bodies (PML-NBs) in the nuclei of normal cells (2). In APL cells, *PML* is fused with the retinoic acid receptor alpha (*RAR*α) gene, resulting in the synthesis of chimeric PML-RARα protein (3). This chimeric protein disrupts PML-NBs leading to their microspeckled dispersion and the loss of their original functions (4). Arsenic trioxide (As_2_O_3_) is the most effective agent in treatment of APL and acts directly on PML. As_2_O_3_ promotes PML-RARα degradation through the SUMO (small ubiquitin-related modifier)-dependent polyubiquitination pathway (5, 6). PML-NBs are dynamic structures. PML maintains the structural stability of PML-NBs and recruits the permanently PML-NB-associated proteins Daxx (death domain-associated protein), SP100 (speckled protein 100), and SUMO, as well as many other proteins transiently residing in the PML-NBs depending on environmental and cellular conditions (2). PML and PML-NBs are involved in multiple biological processes, including tumorigenesis, apoptosis, DNA damage response, and antiviral defense (7). PML and PML-NBs play a vital role in the antiviral response in DNA and RNA virus infection. PML is an intrinsic restriction factor that counteracts viral infection by inhibiting viral replication. However, PML and PML-NBs are targeted and modified by viral proteins to overcome their antiviral activities during infection (8, 9).

Japanese encephalitis virus (JEV) belongs to the family *Flaviviridae*, and is the causative agent of Japanese encephalitis. JEV is a mosquito-borne virus transmitted by *Culex* species, which is prevalent in the eastern, southeastern, and southern regions of Asia (10, 11). Pigs are the main amplifying host for JEV. JEV can cause abortion, stillbirth, and weak piglets in pregnant swine, and neurological symptoms in piglets (12, 13). There is currently no effective therapeutic treatment for JEV, and outbreaks of JEV in pigs are difficult to control, with consequent catastrophic economic losses in the swine industry. PML belongs to the TRIM protein family, some members of which, such as TRIM52, have been shown to affect the replication of JEV. The interaction of JEV non-structural protein 2A (NS2A) and TRIM52 results in degradation of NS2A in a proteasome-dependent manner *via* the E3 ligase activity of TRIM52, which inhibits JEV replication (14). TRIM21 has roles in the regulation of the innate immune response to JEV infection (15). There is accumulating evidence that PML regulates the replication of flavivirus RNA genomes. For example, PML isoforms III and IV, but not the other PML isoforms, repress Dengue virus (DENV) replication. DENV non-structural protein NS5 polymerase complexes with PML III and IV to disrupt PML-NBs, contributing to suppression of the antiviral activity (16). Moreover, PML protein is a driver of anti-Zika virus (ZIKV) intrinsic immunity that inhibits ZIKV infection and replication (17). However, the expression patterns of PML isoforms and the potential roles of PML during JEV infection remain unclear.

Seven PML isoforms generated from a single *PML* gene *via* alternative splicing have been identified in humans. These isoforms share a conserved N-terminal domain but differ in the C-terminal domain. The C-terminal ends of the six PML isoforms (PMLI–PMLVI) contain nuclear localization signals (NLS), and are these isoforms are found in the nucleus, but PMLVII, which has a different C-terminus, is located in the cytoplasm (18). Therefore, the differences in the C-terminal sequences result in different biological functions of each PML isoform (19). Recently, based on the predicted cDNA sequences through analysis of the human and swine *PML* gene sequence, four porcine PML isoforms were cloned by PCR using the cDNAs generated from IFNα-stimulated PK15 cells as templates (20). However, there may be other porcine PML isoforms produced by alternative splicing.

In the present study, we performed molecular cloning and expression analysis of the porcine PML alternative splicing variants during JEV infection. We identified seven alternative splicing variants of porcine PML, all of which contained the same N-terminal sequence but differed in the C-terminal sequences due to alternative splicing. To investigate the role of porcine PML in JEV infection, we analyzed the expression of PML isoforms, Daxx, and SP100 after JEV infection in PK15 cells. The results showed that PML isoforms, Daxx, and SP100 were significantly upregulated during JEV infection. In addition, the number of PML-NBs was increased after JEV infection. These results suggest that porcine PML isoforms may be involved in JEV infection.

## Materials and Methods

### Cell Culture

PK15 cells obtained from the China Center for Type Culture Collection were cultured in Modified Eagle's Medium (MEM; Hyclone, Logan, UT, USA) with 10% fetal bovine serum (FBS; HyClone) and 1% non-essential amino acids (Gibco-BRL Life Technologies, Grand Island, NY, USA). The cells were cultured in an incubator at 37°C and 5% CO_2_.

### 3′ and 5′ Race Assay

Reverse transcription of RNA to make cDNA for Rapid Amplification of cDNA Ends (RACE) assays was performed using a SMARTer® RACE 3′/5′ Kit (Takara, Dalian, China) according to the manufacturer's instructions. The reactions consisted of 1 μL RNA (1 μg/μL), 1 μL 3′-CDS Primer A (12 μM/L), and 10 μL sterile H_2_O (3′ RACE) or 1 μL RNA (1 μg/μL), 1 μL 5′-CDS Primer A (12 μM/L), and 9 μL sterile H_2_O (5′ RACE). The solution was mixed, centrifuged, and incubated at 72°C for 3 min. The tubes were cooled to 42°C for 2 min. For the 5′ RACE cDNA synthesis reaction, 1 μL SMARTer II A Oligonucleotide (24 μM/L) was added. Then, 4 μL 5 × First-Strand Buffer, 0.5 μL 100 μmol/L DTT, 20 U of RNase Inhibitor, and 200 U of SMARTScribe Reverse Transcriptase were added. The reaction was performed in a thermal cycler (T100; Bio-Rad, Hercules, CA, USA) for 90 min at 42°C, followed by heat inactivation for 10 min at 70°C. Samples were stored at −20°C for subsequent use.

The 3′ and 5′ RACE gene-specific primers (GSPs) of the first-step PCR amplification were designed based on the conserved regions of porcine PML sequences in NCBI (GenBank Accession No: XM_001925572.5) (Table 1). The reaction consisted of 10 μL 3′ or 5′ RACE-Ready cDNA, 50 μL 2 × Vazyme LAmp Master Mix (Vazyme, Nanjing, China), 2.5 μL 10 × Universal Primer (UPM) long (10 μM/L), 2.5 μL 3′ Race-GSP (10 μM/L) or 5′ Race-GSP (10 μM/L), and 35 μL ddH_2_O. The PCR consisted of 5 min at 94°C followed by 35 cycles of 94°C for 30 s, 62°C for 30 s, and 72°C for 2 min, with a final step of 72°C for 5 min.

**Table 1:** RACE primer sequences of *PML* gene.

**Names**	**Primer sequence (5^**′**^-3^**′**^)**	**Annealing temperature (^**°**^C)**	**position in cDNA sequence (bp)**
3′ Race-GSP1	CAGTCGGTCGGCGAGTTCCT	62	623–642
3′ Race-GSP2	CAACATCTTCTGCTCCAACCC	62	664–684
3′ Race-GSP3	GTGTCGAGGCGCATCAGT	62	561–578
3′ Race-GSP4	CGGAAGGAAGCCAAATGC	62	290–307
3′ Race-NGSP	CACTCCTGGATACCAGCCACA	62	756–776
5′ Race-GSP	GGATAAGCTCCTCGGTGTCG	62	947–928

If the primary PCR failed to yield the distinct bands of interest, nested PCR was used. The reaction consisted of 30 μL the 3′ RACE first-step PCR product, 50 μL 2 × Vazyme LAmp Master Mix, 2.5 μL 10 × UPM short (10 μM/μL), 2.5 μL 3′ Race-NGSP (10 μM/μL), and 35 μL ddH_2_O.

### Cloning and Sequencing

The products of 5′ RACE first-step PCR or 3′ RACE nested PCR were ligated with pMD19-T vector, and the recombinant vector was transformed into DH5α competent cells. We selected positive clones for sequence analysis. Sequencing results were assembled using DNAStar 8.0 software (DNAStar Inc., Madison, WI, USA). The sequences were compared to the published *PML* gene sequence, and the splicing and retention of exons and introns to obtain full-length cDNA sequences of alternative splicing variants were analyzed.

### Sequence Analysis

DNAMAN 5.0 software (Lynnon Biosoft, Vandreuil, QC, Canada) was used for amino acid sequence alignment. The amino acid sequences of *Homo sapiens* (GenBank Accession No: NP_150241.2), *Mus musculus* (GenBank Accession No: NP_835188.2), *Bos taurus* (GenBank Accession No: XP_005221963.1), and *Capra hircus* (GenBank Accession No: XP_005695206.1) PML were downloaded from NCBI (https://www.ncbi.nlm.nih.gov). The open reading frames (ORFs) of the PML isoforms were analyzed using ORFfinder (https://www.ncbi.nlm.nih.gov/orffinder). Analyses of the conserved domains were performed using CD-Search (https://www.ncbi.nlm.nih.gov/Structure/cdd/wrpsb.cgi) and SMART (http://smart.embl-heidelberg.de). The NLS was predicted using NucPred (https://nucpred.bioinfo.se/nucpred). The small ubiquitin-like modifier modification (sumoylation) sites were predicted using SUMOsp 2.0 software (http://sumosp.biocuckoo.org).

### Plasmid Construction and Transfection

PML isoforms were amplified from cDNA of PK15 cells with the same forward primer and corresponding reverse primers (Table 2). The amplified fragments (PML-1/2, PML-3, PML-4/5, PML-6, and PML-7) were inserted into the pEGFP-C1 vector. PK15 cells were cultured in 12-well plates. The five PML recombinant eukaryotic vectors were transfected into the cells using Lipofectamine 3000 transfection reagent (Invitrogen, Carlsbad, CA, USA) according to the manufacturer's instructions. At 36 h post-transfection, cells were harvested for RT-qPCR and fluorescence analysis.

**Table 2:** Primers used for porcine PML isoforms cloning.

**Names**	**Primer sequence (5**′~**3^**′**^)**	**Size (bp)**
PML	F: *C*CCAAGCTTCCATGCAGCAGGAACCGGCA	
PML-1/2	R: *C*GCGGATCCTCAGCTCTCCTGGGAAGCCCT	2,625
PML-3	R: *C*GCGGATCCTTAGAGGCTTGTCTGCGGGGTG	2,433
PML-4/5	R: *C*GCGGATCCTCACTGCCTTGCTGGCA	1,809
PML-6	R: *C*GCGGATCCTTATTGAGTAGCCACACCTGCCAGGGCC	1,569
PML-7	R: *C*GCGGATCCTCAGGGGTGCAGGTCAA	1,263

*F, forward primer; R, reverse primer. The enzyme cleavage site and protective base were underlined*.

### Immunofluorescence Analysis

PK15 cells were washed three times and fixed with 4% paraformaldehyde for 20 min at room temperature. Then the cells were permeabilized with 0.25% Triton X-100 for 15 min. After blocking with PBS containing 5% bovine serum albumin (BSA), the cells were stained with rabbit anti-PML polyclonal antibody (1:100; GeneCreate, Wuhan, China) at room temperature for 1 h. After washing three times with PBS, the cells were incubated with Alexa Fluor 488-conjugated goat anti-rabbit antibody (A-11008, 1:1,000; Invitrogen, Carlsbad, CA, USA). The nuclei were stained with 4′,6-diamidino-2-phenylindole (DAPI).

### Viral Infection

PK15 cells were cultured in 12-well cell culture plates, and infected with JEV strain SA14–14–2 (MOI = 1) diluted in MEM (21). The infected cells were washed twice with PBS after 1 h of adsorption, and then maintained in MEM with 2% FBS. The infected cells were collected for RNA isolation at the indicated time points after infection.

### RT-qPCR Assay

Total RNA was extracted using TRIzol reagent (Invitrogen). Total RNA (1 μg) was used to synthesize cDNA with an RT-PCR reagent kit (CWBiotech, Beijing, China). Quantitative analysis was performed using SYBR Premix Ex Taq II (Takara) on a CFX96 Touch instrument (Bio-Rad). The sequences of the qPCR primers used in the present study are listed in Table 3. The following PCR cycling conditions were used: denaturation at 95°C for 2 min, followed by 40 cycles of 95°C for 5 s, 60°C for 30 s, and 72°C for 20 s, and then melting curve analysis. Relative expression levels were calculated using the 2^−Δ*ΔCt*^ method and *GAPDH, ACTB, 18srRNA*, and *RPL32* as reference genes (22).

**Table 3:** The sequences of RT-qPCR primers.

**Names**	**Primer sequence (5**′~**3**′**)**	**Annealing temperature (^**°**^C)**	**Size (bp)**
*JEV-E*	F: GTCCATAGGGAGTGGTTTCA R: CCTTTCAGAGCCAGTTTGTC	60	257
*PML*	F: CGGAAGGAAGCCAAATGC R: TATCCAGGGCCTGCGTGT	60	136
*PML-1/2*	F: CCACAAGAGGGCCTGAAGAA R: TGTCGAAGTAGGTGCCCAGA	60	109
*PML-3,4/5,6*	F: CCTCTGGGCCTCTGCCGGGATG R: GGCCTGGGAGCAGCAGAGTCCTTGC	60	60
*PML-7*	F: GGACAGGAAGCTCGCTCAT R: CAGGCAAGCACCCAACAT	60	127
*Daxx*	F: GCCTGATACCTTCCCTGACTA R: TGACAGCCGAAGTTGTAGATG	60	180
*SP100*	F: TAGAAAGGCAACAAGCAAA R: ATATCCACCAGATAGACAGAA	60	196
*GAPDH*	F: GGACTCATGACCACGGTCCAT R: TCAGATCCACAACCGACACGT	60	220
*ACTB*	F: TGCGGGACATCAAGGAGAA R: AGGAAGGAGGGCTGGAAGA	60	175
*18srRNA*	F: TGAGAAACGGCTACCACATCC R: GGGCCTCGAAAGAGTCCTG	60	108
*RPL32*	F: CGGAAGTTTCTGGTACACAATGTAA R: TGGAAGAGACGTTGTGAGCAA	60	94

### Western Blotting

Total proteins of JEV-infected and uninfected PK15 cells were extracted using RIPA lysis buffer with phosphatase and protease inhibitor (CWBiotech) respectively. Then the cell lysates were separated by SDS-PAGE and transferred onto polyvinylidene difluoride (PVDF) membranes (Millipore, Billerica, MA, USA). After blocking with 5% non-fat milk for 1 h, the membranes were incubated with rabbit anti-PML polyclonal antibody (1:1,000; GeneCreate) and rabbit anti-JEV (NS3) polyclonal antibody (GTX125868, 1:5,000; GeneTex, Irvine, CA, USA) overnight at 4°C. After washing three times, the membranes were incubated with horseradish peroxidase (HRP)-conjugated goat anti-rabbit secondary antibody (A21020; 1:10,000; Abbkine, Wuhan, China) for 1 h at room temperature. Then the membranes were imaged using a Tanon 5200 system (Biotanon, Shanghai, China) after treatment with HRP Substrate (Merck Millipore, Darmstadt, Germany). β-Actin was used as an internal control.

### Statistical Analysis

All data are presented as the mean ± SEM of three independent experiments. Statistical significance was assessed using Student's *t* test. In all analyses, *p* < 0.05 was taken to indicate statistical significance.

## Results

### Identification and Cloning of 3′ and 5′ Porcine *PML* Gene cDNA Sequences

**Figure 1:**
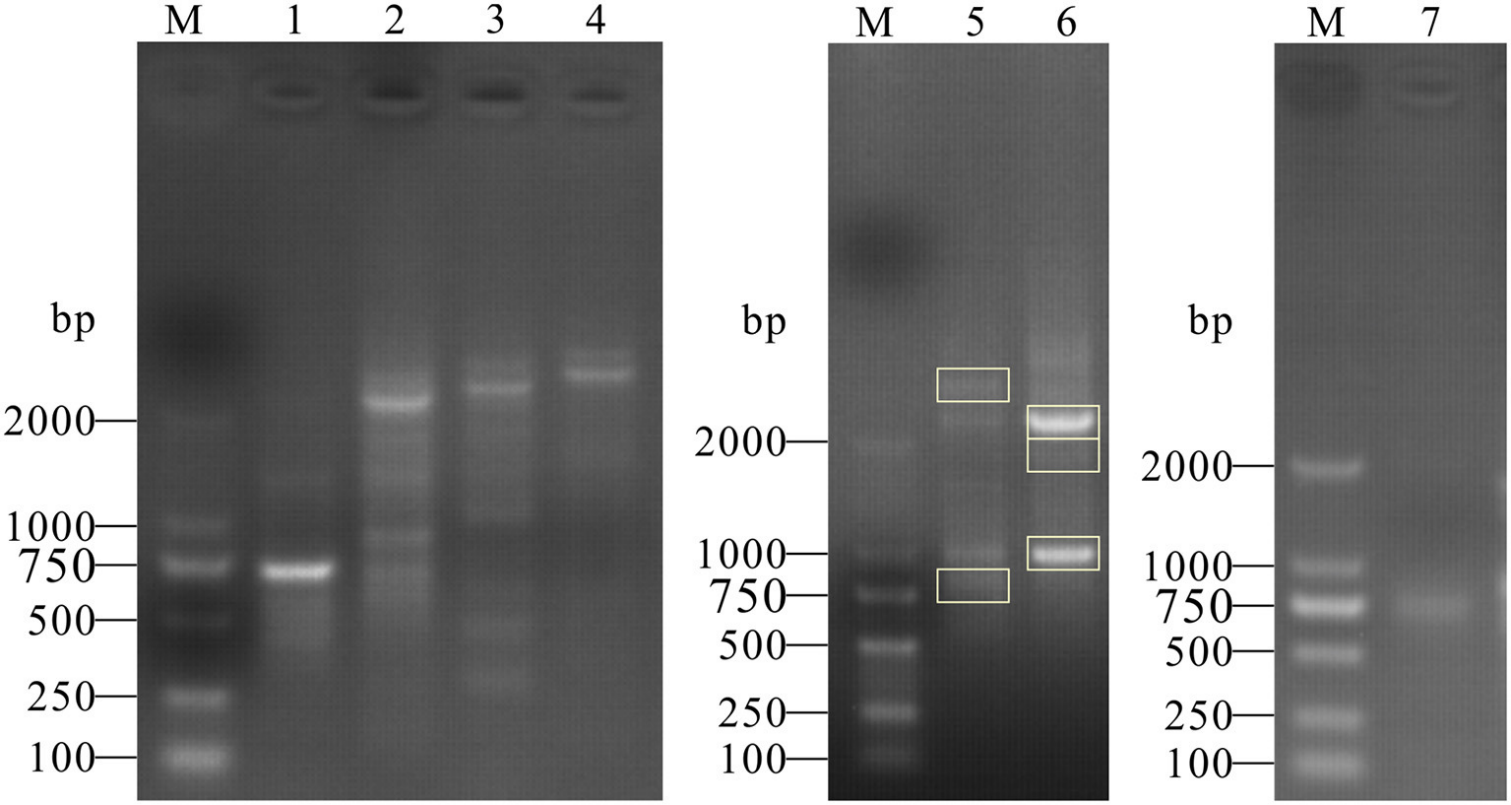
PCR products of 3′ and 5′ RACE of the porcine *PML* gene. M: DL2000 Marker; the marker fragment sizes were 100, 250, 500, 750, 1,000, and 2,000 bp. Lanes 1, 2, 3, 4: PCR products of the first 3′ RACE cycle. Labels on the lanes represent different groups of 3′ RACE primers (3′ Race-GSP1, GSP2, GSP3, and GSP4). Lanes 5, 6: PCR products of the second 3′ RACE cycle (3′ Race-GSP2 and 3′ Race-GSP3 first-step PCR products as 3′ RACE-Ready cDNA), and gel bands in the rectangular box were selected for cloning analysis. Lane 7: PCR products of the first 5′ RACE cycle.

**Figure 2:**
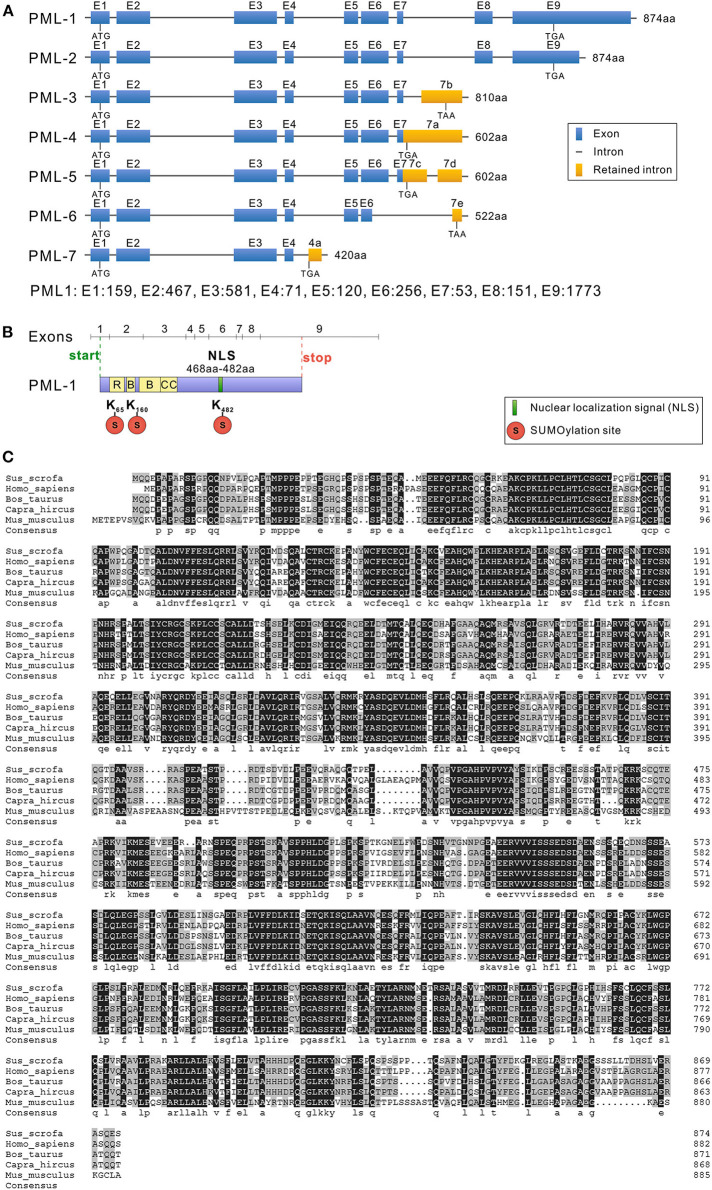
Structure and sequence analysis of porcine PML isoforms. **(A)** The lengths of seven PML isoforms (PML-1 to PML-7) encoded by the different mRNA variants and their exon compositions from the translational start codons to the stop codons of the mRNA are shown. The full-length genomic sequence of PML includes nine exons (1–9). Alternative splicing of the *PML* gene leads to seven mRNA variants. Exon length: exon 1, 159 bp; exon 2, 467 bp; exon 3, 581 bp; exon 4, 71 bp; exon 5, 120 bp; exon 6, 256 bp; exon 7, 53 bp; exon 8, 151 bp; exon 9, 1,773 bp. **(B)** Conserved domain analysis of PML-1. **(C)** *Sus scrofa, Homo sapiens, Bos taurus, Capra hircus*, and *Mus musculus* PML-1 amino acid sequence alignment was performed with DNAMAN 5.0 software. Identical amino acids are indicated by a black background, and amino acids with substitutions are shown in light gray.

cDNA for RACE assay was amplified from PK15 cells by RT-PCR. The products of the first cycle (using 3′ Race-GSP and 5′ Race-GSP) and the second cycle of nested PCR (using 3′ Race-NGSP) were analyzed by agarose gel electrophoresis (Figure 1). As shown in Figure 1, specific bands were obtained from 3′ RACE nested PCR and 5′ RACE first-step PCR, and the PCR products were cloned into the pMD19-T vector. After transformation into DH5α cells, positive clones were selected and sequenced. From the sequencing and alignment results, we identified seven types of PML cDNA clone, which we designated, from longest to shortest, as PML-1, PML-2, PML-3, PML-4, PML-5, PML-6, and PML-7 (Figure 2A).

### Structural Analysis of Seven Alternative Splicing Variants of Porcine *PML* Gene

Sequencing results were assembled using DNAStar 8.0 software (DNAStar Inc.), and the gene structures of the seven cloned different isoforms of the porcine *PML* gene are shown in Figure 2A. The full-length mRNA sequence of PML (PML-1) was 3,659 bp in length, and consisted of nine exons. The coding DNA sequence (CDS) of PML-1 was 2,625 bp and encoded 874 amino acids. The 3′ untranslated region (UTR) was 1,009 bp, and the 5′ UTR was much shorter at only 25 bp. PML-2 also encoded a product of 874 amino acids, but the 3′ UTR of PML-2 only retained 400 bp of the PML-1 3′ UTR. PML-3 contained exons 1–7 and retained part of the intron 7 sequence (7b: 641–1,788 bp), and encoded a product of 810 amino acids. PML-4 contained exons 1–7 and retained the partial sequence of intron 7 (7a: 1–1,785 bp), and encoded a product of 602 amino acids. PML-5 also contained exons 1–7 and retained part of the intron 7 sequence (7c: 1–434 bp, 7d: 1,245–1,788 bp). PML-6 contained exons 1–5 and retained a partial sequence of exon 6 (at 1–109 bp) and intron 7 (7e: 1,685–1,781 bp). PML-6 encoded a product of 522 amino acids. PML-7 contained exons 1–4 and retained a partial sequence of intron 4 (4a: 540–865 bp), and it encoded a product of only 420 amino acids. The PML-1 and PML-3 alternative splicing variants cloned here were the same as sPML-I and sPML-II cloned in a previous study (20).

All of the PML isoforms shared exons 1–4 and contained the same N-terminal domain but different C-terminal domains. The *PML* gene is a member of the *TRIM* gene family, and is also called TRIM19. Therefore, the first three exons of the PML isoforms contained the RING zinc finger, two B-box domains, and a coiled-coil motif (Figure 2B). Like other mammals, porcine PML-1 to PML-6 proteins included three sumoylation sites (at residues 65, 160, and 482), and PML-7 contained two sumoylation sites (at residues 65 and 160). Exon 6 encoded the NLS (Figure 2B). Therefore, six nuclear PML isoforms PML-1 to PML-6 were localized in the nucleus, while PML-7 was localized in the cytoplasm.

### Sequence Alignment

To understand the evolutionary characteristics of the *PML* gene, we selected the porcine PML-1 protein and the longest PML isoform of human (*H*. *sapiens*), cattle (*B*. *taurus*), goat (*C*. *hircus*), and mouse (*M*. *musculus*) for amino acid sequence alignment using DNAMAN 5.0 software (Figure 2C). The amino acid sequence of porcine PML-1 showed 76.21, 77.17, 77.05, and 61.78% identity those of human, cattle, goat, and mouse, respectively. The identity of the *PML* gene varied highly at the N- and C-terminals. The RBCC motif and three sumoylation sites were very highly conserved. These results indicate that the PML protein was moderately conserved in these species and the functional domains were highly conserved.

### Effects of Porcine PML Isoforms on PML-NB Distribution

**Figure 3:**
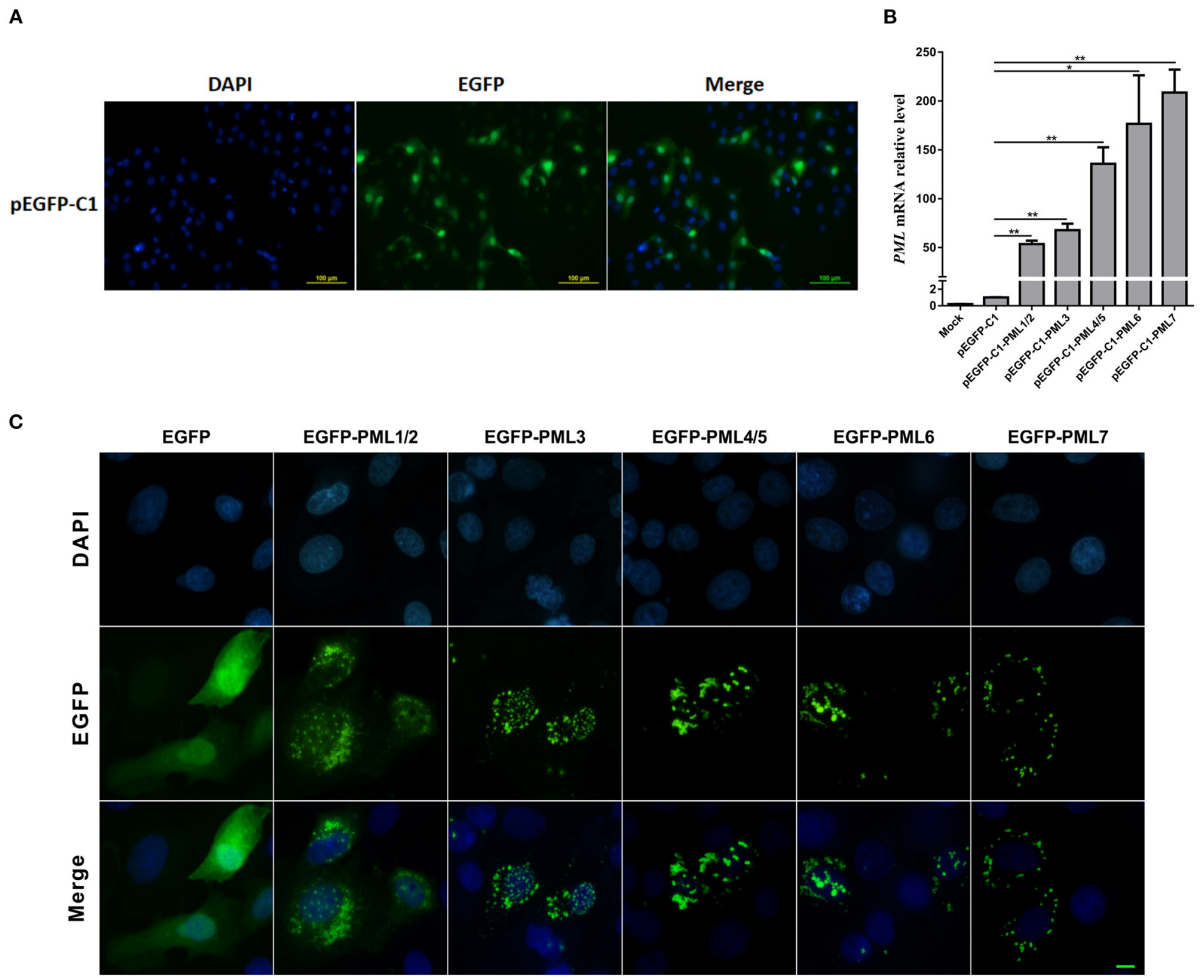
Subcellular distribution of PML-NBs in PK15 cells after transfection of pEGFP-C1-PML isoform recombinant plasmids. PK15 cells were transfected with pEGFP-C1-PML1/2, PML3, PML4/5, PML6, and PML7 plasmids for 36 h. **(A)** Fluorescence analysis of enhanced green fluorescent protein (EGFP) in PK15 cells transfected with the empty vector (pEGFP-C1). Nuclei were stained with DAPI (blue). **(B)** RT-qPCR analysis of the expression levels of PML isoforms after transfection with pEGFP-C1-PML1/2, PML3, PML4/5, PML6, and PML7. *p < 0.05, **p < 0.01. **(C)** The subcellular distribution of PML-NBs in PK15 cells overexpressing different PML isoforms. Scale bar: 10 μm.

PML is a critical component of PML-NB formation, and the PML-NBs exhibit a dot-like structure in immunofluorescence images (23). To determine the roles of the seven PML isoforms in the formation and distribution of PML-NBs, PML isoforms were overexpressed in PK15 cells by transfection with the recombinant eukaryotic vectors pEGFP-C1-PML1/2, PML3, PML4/5, PML6, and PML7, respectively. The transfection efficiency was determined by the expression of enhanced green fluorescent protein (EGFP). The transfection efficiency reached approximately 30%, as demonstrated by EGFP expression (Figure 3A). The expression of PML isoforms was examined by RT-qPCR. Compared to that of cells transfected with pEGFP-C1 as an empty vector control, PML was significantly overexpressed in pEGFP-C1-PML isoform-transfected cells (Figure 3B). Further, the typical PML-NBs could be observed after overexpression of the five PML isoforms in PK15 cells (Figure 3C). PML-1/2, PML-3, PML-4/5, and PML-6 formed typical PML-NBs in the nucleus, and the EGFP-PML fusion proteins were also expressed in the cytoplasm. However, PML-7 formed PML-NBs that were only observed in the cytoplasm.

### Expression of PML Isoforms During JEV Infection

**Figure 4:**
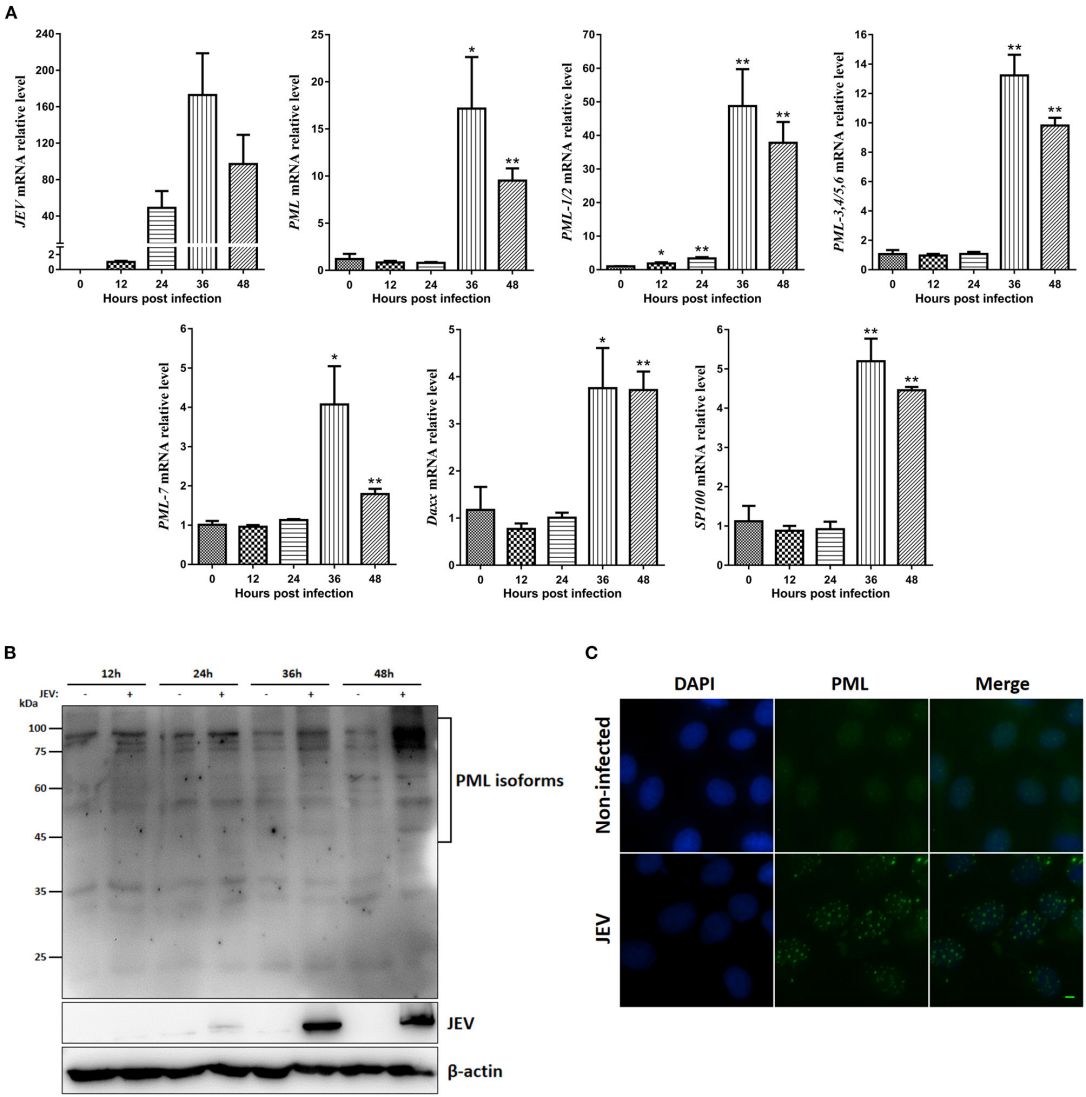
Expression of PML isoforms, *Daxx, SP100*, and PML-NBs during JEV infection. **(A)** *JEV-E*, PML isoform, *Daxx*, and *SP100* mRNA levels were examined by RT-qPCR. *GAPDH, ACTB, 18srRNA*, and *RPL32* levels were used as loading controls, and the relative expression levels were expressed as fold change relative to mock-infected cells. *p < 0.05, **p < 0.01. **(B)** Western blotting analysis of PML protein expression during JEV infection at the indicated time points. **(C)** PK15 cells were infected with JEV for 36 h, and then immunofluorescence staining of PML protein (green) was performed using anti-PML antibody. Nuclei were stained with DAPI (blue). Non-infected cells were used as controls. Scale bar: 10 μm.

PML and PML-NBs play important roles in the innate immune response and antiviral defense (9). To investigate the porcine *PML* gene expression patterns after JEV infection, PK15 cells were infected with JEV, and the cells were harvested at the indicated time points. Viral replication was monitored by RT-qPCR, and the viral mRNA in PK15 cells peaked at 36 h post-infection (hpi) (Figure 4A). The expression of PML isoforms and PML-NB-associated genes (*Daxx* and *SP100*) were examined (Figure 4A). JEV infection significantly upregulated the mRNA expression of PML isoforms, *Daxx* and *SP100* at 36 and 48 hpi. Among them, the *PML-1/2* isoform expression was upregulated by 48-fold at 36 hpi. The PML protein levels were examined by Western blotting analysis using a polyclonal antibody against the N-terminal shared amino acids of the PML isoforms (Figure 4B). PML isoforms were upregulated at 36 and 48 hpi. To further determine the characteristics of the PML-NBs in JEV-infected PK15 cells, the cells were infected with JEV for 36 h and then we performed immunofluorescence analysis of the infected cells using anti-PML polyclonal antibody (Figure 4C). Compared to non-infected cells, the number of PML-NBs was significantly increased in JEV-infected cells, with approximately 20–30 PML-NBs in each cell. These results suggest that PML and PML-NBs may play important roles in JEV infection.

## Discussion

PML has multiple roles in biological processes, including antiviral defense (9). The human *PML* gene contains nine exons and expresses several mature transcripts *via* alternative splicing, yielding at least seven main PML isoforms (24). In the present study, we cloned the porcine *PML* gene and found seven PML mRNAs by 3′ and 5′ RACE assays. As alternative splicing of the *PML* gene primarily occurred at the C-terminus, PCR primers were designed to amplify the C-terminus of PML transcripts with a RACE kit according to the manufacturer's instructions. We obtained several fragments by gel electrophoresis, and five fragments were selected for sequence analysis. We ultimately obtained seven mature transcripts using sequence assembly with the 5-terminal amplified fragment. As we only selected five fragments with obviously amplified bands *via* gel electrophoresis for sequencing, additional PML isoforms may have been identified had we sequenced all of the amplified fragments.

We named the longest PML mRNA with nine exons PML-1. Notably, PML-2 contained a shorter exon 9 but encoded the same protein as PML-1. That is, PML-2 had a shorter 3′ UTR compared to PML-1. More miRNAs may regulate PML-1 *via* the 3′ UTR compared to PML-2. Alternative splicing of PML-3 to PML-6 was located in intron 7 and excluded exons 8 and 9. This feature of splicing patterns is similar to human PML. Human PML-II, PML-III, PML-V, and PML-VI also retain partial sequences of intron 7 (25). These results indicate that the splicing patterns of the *PML* gene were conserved in different species. Similar to PML-2, PML-4 and PML-5 encoded the same protein with a variable 3′ UTR. These two mRNAs may participate in different signal regulation processes. These PML isoforms all had the RBCC motif, and PML is a member of the TRIM family. PML-1 to PML-6 had the NLS, and they were nuclear proteins. PML-7 lacked the NLS, and was likely cytoplasmic. Similarly, PML-I to PML-VI in humans are present in the nucleus, while PML-VII is a cytoplasmic form (23, 25). PML sumoylation is crucial for the formation of PML-NBs (26). There were three sumoylation sites in PML-1 to PML-6, which were highly conserved in different species based on protein sequence alignment analysis. However, further studies are required to determine the functional roles of sumoylation of porcine PML.

PML protein was mainly localized in the PML-NBs in cells overexpressing each of the seven human isoforms (16, 27). This study identified the subcellular distribution of porcine PML isoforms by overexpressing different EGFP-fused PML isoforms. PML-1/2, PML-3, PML-4/5, and PML-6 contained the NLS, and the typical PML-NBs were found in the nucleus. These PML isoforms were also localized in the cytoplasm. The cellular localization of PML-NBs is dynamic due to the shuttling of PML-associated components, which is determined by specific PML isoforms (28, 29). We speculate that these isoforms are the critical shuttle proteins in the PML-NB reformation process. In addition, PML-7 was localized in the cytoplasmic PML-NBs due to its loss of the NLS (30). The NLS is essential for PML-NBs nuclear localization. The PML-NBs were only distributed in the cytoplasm when the NLS sequence was mutated (27).

There is accumulating evidence that the PML protein and PML-NBs have antiviral effects. For example, PML-NBs epigenetically modify the genomes of human herpes simplex virus type 1 (HSV-1) and human cytomegalovirus (HCMV), resulting in silencing of the viral genomes during the early stages of virus infection (9). PML-IV protein interacts with varicella-zoster virus (VZV) capsid protein and impedes the release of virus particles from the nuclei (31). Another study showed that only PML-III and PML-IV inhibited vesicular stomatitis virus (VSV), and PML-IV promoted the expression of IFN-β by enhancing IRF3 activity (32). The expression levels of PML isoforms, *Daxx*, and *SP100* were significantly upregulated at 36 and 48 hpi. These expression trends were consistent with the viral growth curve, and the JEV viral particles peaked at 36 and 48 hpi (33). These results suggest that the PML-NB-associated proteins (PML isoforms, Daxx, and SP100) may play important roles in defense against JEV infection. In addition, the number of PML-NBs was significantly increased after JEV infection, suggesting that the components and morphology of PML-NBs vary in response to virus infection (9, 34). The results of Western blotting analyses showed that several PML isoforms were induced during JEV infection. The functions of the seven human PML isoforms are different, and further studies are required to determine the potential roles of these porcine PML isoforms and PML-NBs in JEV infection.

In summary, we cloned seven porcine PML alternative splicing variants, whose isoforms shared a common N-terminus and differed in the C-terminus. These seven alternative splicing variants encoded five proteins. Sequence alignment analysis showed that the RBCC and three sumoylation sites were highly conserved in different species. The expression levels of PML isoforms, *Daxx*, and *SP100* were significantly upregulated, and the number of PML-NBs was increased during JEV infection. Further studies are required to determine the functions of these PML isoforms in JEV infection.

## Data Availability Statement

The datasets generated and analyzed are available in the GenBank repository with the accession numbers PML-1 (MW490598), PML-2 (MW490599), PML-3 (MW490600), PML-4 (MW490601), PML-5 (MW490602), PML-6 (MW490603), and PML-7 (MW490604).

## Author Contributions

SY conceived and designed the experiments. JZ, ZC, and ZD performed the experiments. XZ, HW, and XL analyzed the data. AZ and SY helped to write the manuscript. All authors read and approved the final manuscript.

## Funding

This work was supported by the Zhejiang Provincial Natural Science Foundation of China (LY19C170002, LY19C170003), the Zhejiang Provincial Key Research and Development Program (2021C04034), and the National Undergraduate Training Program for Innovation and Entrepreneurship (201910341043).

## Conflict of Interest

The authors declare that the research was conducted in the absence of any commercial or financial relationships that could be construed as a potential conflict of interest.

## Publisher's Note

All claims expressed in this article are solely those of the authors and do not necessarily represent those of their affiliated organizations, or those of the publisher, the editors and the reviewers. Any product that may be evaluated in this article, or claim that may be made by its manufacturer, is not guaranteed or endorsed by the publisher.

## Abbreviations

As_2_O_3_: arsenic trioxide
Daxx: death domain-associated protein
DENV: Dengue virus
GSP: gene-specific primer
hpi: hours post-infection
JEV: Japanese encephalitis virus
NGSP: nested gene-specific primer
NLS: nuclear localization signal
PML: promyelocytic leukemia
PML-NBs: promyelocytic leukemia nuclear bodies
RARα: retinoic acid receptor alpha
RT-qPCR: quantitative reverse transcription PCR.
SP100: speckled protein 100
SUMO: small ubiquitin-related modifier
TRIM: tripartite motif
UTR: untranslated region
ZIKV: Zika virus.
